# Simultaneous Intake of *Euglena gracilis* and Vegetables Exerts Synergistic Anti-Obesity and Anti-Inflammatory Effects by Modulating the Gut Microbiota in Diet-Induced Obese Mice

**DOI:** 10.3390/nu11010204

**Published:** 2019-01-21

**Authors:** Ran Okouchi, Shuang E, Kazushi Yamamoto, Toshikuni Ota, Kentarou Seki, Mayumi Imai, Ryuki Ota, Yuta Asayama, Ayaka Nakashima, Kengo Suzuki, Tsuyoshi Tsuduki

**Affiliations:** 1 Laboratory of Food and Biomolecular Science, Graduate School of Agriculture, Tohoku University, Sendai 981-8555, Japan; orchid5416@gmail.com (R.O.); eshuang0@yahoo.co.jp (S.E.); kazushi.yamamoto.t1@dc.tohoku.ac.jp (K.Y.); 2 Takeda Consumer Healthcare Company Limited, Chiyoda-ku, Tokyo 100-0005, Japan; toshikuni.oota@takeda.com (T.O.); kentarou.seki@takeda.com (K.S.); mayumi.imai@takeda.com (M.I.); ryuki.ota@takeda.com (R.O.); 3 Euglena Co., Ltd, Minato-ku, Tokyo 108-0014, Japan; asayama@euglena.jp (Y.A.); nakashima@euglena.jp (A.N.); suzuki@euglena.jp (K.S.)

**Keywords:** *Euglena gracilis*, gut microbiota, inflammation, paramylon, vegetable, visceral fat

## Abstract

We determined whether the anti-obesity effect provided by the consumption of *Euglena gracilis* (Euglena), which is rich in insoluble dietary fiber, could be enhanced by the co-consumption of vegetables with an abundance of soluble dietary fiber. Nine-week-old male C57BL/6J mice were divided into five groups as follows: group 1 received a normal diet, group 2 received a high-fat diet, and groups 3, 4, and 5 received high fat diets containing 0.3% paramylon, 1.0% Euglena, or 1.0% Euglena plus 0.3% vegetables (barley leaf, kale, and ashitaba), respectively. Mice were fed ad libitum until 18 weeks of age. Euglena intake significantly reduced visceral fat accumulation in obese mice, and co-consumption of vegetables enhanced this effect. Consumption of Euglena with vegetables reduced adipocyte area, suppressed the expression of genes related to fatty acid synthesis, upregulated genes related to adipocyte lipolysis, and suppressed serum markers of inflammation. Notably, we also observed an increase in the fraction of short-chain fatty acid-producing beneficial bacteria, a reduction in harmful bacteria that cause inflammation, and an increase in short-chain fatty acid production. Therefore, the co-consumption of vegetables enhanced the anti-obesity and anti-inflammatory effects of Euglena, likely by modulating the gut microbiota composition.

## 1. Introduction

In recent years, the rapid Westernization of eating habits in Japan has led to an increased risk of obesity and other lifestyle diseases [1]. Imbalance in various nutrients is a characteristic of the contemporary diet [2]. Consuming a balance of these nutrients is thus considered important for the prevention of lifestyle diseases. Recently, *Euglena gracilis* (Euglena) has gained attention as a new health food, as it is rich in vitamins, minerals, and other nutrients [3], and also contains the insoluble fiber paramylon as a characteristic component. Paramylon, a triple-helical, high-polymer substance composed of linear β-1,3-glucans, is known to prevent obesity and diabetes [4,5]. Insoluble fiber absorbs water easily, increasing stool quantities and easing bowel movements. It also functions to prevent colorectal cancer by eliminating harmful substances from the body [6]. Insoluble fiber includes celluloses, hemicelluloses, and lignins, whereas soluble fiber comprises pectins, gums, and reserved polysaccharides [7,8]. Soluble fiber inhibits the absorption of glucose and fats from the intestinal tract and functions to lower blood sugar and lipid levels [9]. Through fermentation by intestinal microbiota, soluble fiber also yields the production of short-chain fatty acids, which are used by large intestinal mucosal cells as an energy source to enhance their protective effects on the intestine [10]. Paramylon and other fiber types contained in Euglena are almost all insoluble.

Many studies have been performed on Euglena and paramylon, but few studies have explored ways to increase their efficacy. Accordingly, we hypothesized that a balanced fiber intake, focusing on vegetables that are rich in soluble fiber, could enhance the efficacy of Euglena. Soluble fiber in vegetables, such as barley leaf, kale, and ashitaba, has been found to delay excess nutrient absorption in the intestine and promote short-chain fatty acid production through intestinal fermentation, thereby improving the intestinal environment [11]. Our previous study showed that Euglena intake could significantly decrease visceral fat in normal mice, which was further augmented by simultaneous vegetable consumption [12]. Further, we reported that the simultaneous consumption of Euglena and vegetables decreased adipocyte area, suppressed gene expression related to fatty acid synthesis, increased gene expression related to adipocyte proliferation and lipolysis, and suppressed serum inflammation parameters. Mechanistically, Euglena increased short chain-fatty acid-producing beneficial bacteria and decreased harmful bacteria that cause inflammation in the intestinal microbiota and enhanced short-chain fatty acid production. As the simultaneous consumption of Euglena and vegetables yielded beneficial effects in normal mice, we expected that this would also have an anti-obesity effect. Thus, in this study, we examined the effect of Euglena and vegetables using a mouse model of high-fat diet-induced obesity.

## 2. Materials and Methods 

### 2.1. Test Diets

Test diets to test the effect of Euglena combined with vegetables on diet-induced obesity included paramylon, Euglena (spray-dried powder of *Euglena gracilis*), and vegetables [mixture of spray-dried powder of *Euglena gracilis*, barley leaf (*Hordeum vulgare*), kale (*Brassica oleracea var. acephala*), and ashitaba (*Angelica keiskei*)]. Diet-enriching samples were provided by Takeda Consumer Healthcare Company Limited and euglena Co., Ltd. Paramylon is an indigestible dietary fiber that is a major physiologically active substance in Euglena [3]. Barley leaf, kale, and ashitaba all contain large amounts of water-soluble dietary fiber [11]. Paramylon, Euglena, or Euglena + vegetable samples were mixed with a high-fat diet (Western Diet-D12079BM, Research Diet Inc.; Table 1). Five test diets were prepared as follows: Normal diet (control; 98121701M, Research Diet Inc.); high-fat diet; High-Fat + Paramylon—containing 0.3% paramylon (as Euglena contains 30% paramylon [3,4,5,12], 0.3% functionally corresponded to 1% whole Euglena content); high-fat + Euglena diet—containing 1.0% Euglena; and high-fat + Euglena + vegetables—containing 1.0% Euglena and 0.3% vegetables (ND, HD, HDP, HDE, or HDE + V groups, respectively). Euglena and vegetable intakes were decided by referring to the previous report, taking into account the human intake amount [12]. The composition of each test diet is shown in Table 1. The energy content per 100 g of each test diet was 389, 465, 464, 461, or 459 kcal. Energy was calculated using the modified Atwater method (4 kcal of protein, 9 kcal of lipid, 4 kcal of carbohydrate per g) [13,14].

### 2.2. Animals

All animal procedures were performed in accordance with the Animal Experiment Guidelines of the Tohoku University, and the animal protocol was approved by the Animal Use Committee at the Tohoku University (2016AgA-009) [15,16]. Male C57BL/6J mice (9-weeks-old) were obtained from Clea Japan (Tokyo, Japan). Mice had access to respective diets and distilled water ad libitum in a temperature- and humidity-controlled room with a 12/12-h light/dark cycle. After acclimatization to the normal diet for one week, 50 mice were randomly divided into five groups. Each group comprised 10 mice, divided into two cages. Mice were fed test diets for eight weeks. At 18 weeks of age, after a 12-hour fast, mice were weighed, and blood samples were collected after decapitation. To obtain serum, blood was centrifuged (900× *g*, 5 °C, 15 min). Brain, heart, lung, liver, pancreas, spleen, kidney, white adipose, and cecum tissues were removed and weighed. Serum and organs were stored at −80 °C until use.

### 2.3. Histological Analysis

For histological analysis, epididymal adipose tissue was fixed in 10% formalin and embedded in paraffin [17,18]. Vertical sections (5-μm) were cut, mounted on a glass slide, stained with hematoxylin and eosin (HE), and observed using a microscope (BZ-9000; Keyence, Osaka, Japan). The mean area of adipocytes was calculated in each group [17,18].

### 2.4. mRNA Expression Analysis

For real-time quantitative reverse transcriptase PCR (qRT-PCR), total RNA was isolated from epididymal adipose tissue using an RNeasy Mini Kit (Qiagen, Valencia, CA, USA) [19], eluted with 40 μL of RNase-free water, and stored at −80 °C until use. To quantify gene expression, mRNA levels for fatty acid synthase (*Fans*), glucose-6-phosphate dehydrogenase X-linked (*G6pdx*), malic enzyme (*Me*), peroxisome proliferator-activated receptor gamma (*Pparg*), sterol regulatory element binding factor 1 (*Srebf1*), and -actin (*Actb*) in epididymal adipose tissue were determined using a Thermal Cycler Dice Real Time System® (Takara Bio, Otsu, Japan). This system allowed real-time quantitative detection of PCR products by measuring the increase in fluorescence caused by binding of SYBR green to double-stranded DNA [20]. In brief, cDNA was generated using Prime Script® RT Master Mix (Perfect Real Time; Takara Bio, Otsu, Japan) from epididymal adipose tissue total RNA. cDNA was subjected to PCR amplification using SYBR® Premix Ex TaqTM (Perfect Real Time; Takara Bio) and gene-specific primers for *Fas*, *G6pdx*, *Me*, *Pparg*, *Srebf1*, and *Actb* (Table 2). PCR amplification was performed as follows: Activation at 95 °C for 10 s, followed by 40 cycles at 95 °C for 5 s and 60 °C for 31 s, and dissociation at 95 °C for 15 s, 60 °C for 30 s, and 95 °C for 15 s for each gene. Melting curve analysis was performed following each reaction to confirm the presence of a single reaction product. The threshold cycle (Ct) represented the PCR cycle at which an increase in reporter fluorescence above baseline signals was first detected. mRNAs levels were normalized to those of *Actb* in test samples.

### 2.5. Serum and Liver Biochemical Analyses

Biochemical analyses of serum and liver samples were performed as described previously [21,22]. Serum and liver triacylglycerol, total cholesterol (TC), serum phospholipid, glucose, alanine aminotransferase, and aspartate transaminase levels were measured using commercial enzyme kits (Wako Pure Chemical, Osaka, Japan). Serum insulin and leptin were determined using an ELISA kit (Morinaga Institute of Biological Science, Yokohama, Japan). Liver phospholipids were measured using the procedure of Rouser et al [23]. Serum IL-1β and IL-6 were determined using an ELISA kit (BD Biosciences, Franklin Lakes, NJ, USA). Serum and liver thiobarbituric acid reactive substances (TBARS) were measured as described previously [24]. A microplate reader (Infinite F200, Tecan Japan Co., Ltd., Kanagawa, Japan) was used for absorbance measurements.

### 2.6. Analysis of Gut Microbiota

Intestinal bacterial DNA was extracted from mouse stool samples, and the gene encoding 16S rRNA was amplified by PCR. The composition of intestinal bacteria in feces was examined by meta 16S analysis as described previously [13,25]. Mouse feces was collected daily during the last week of the study period, and DNA was extracted from the recovered feces and pooled for each group. DNA extraction from mouse feces was performed using a QIAmp DNA Stool Mini Kit (Qiagen, Hilden, Germany). PCR-based 16S rRNA gene amplicon sequencing was performed using 10 ng DNA, 10 µM of each barcoded forward and reverse primer, 2× Gflex PCR Buffer (Mg^2+^, dNTP plus) (Takara Bio) and Tks Gflex DNA Polymerase (Takara Bio) in a volume of 25 µL. To target 16S rRNA variable regions 3 and 4 (V3 and V4), the forward primer 341F (5-TCGTCGGCAGCGTCAGATGTGTATAAGAGACAGCCTACGGGNGGCWGCAG-3) and reverse primer 806R (5-GTCTCGTGGGCTCGGAGATGTGTATAAGAGACAGGGACTACHVGGGTWTCTAAT-3) were used at 94 °C for 1 min followed by 28 cycles of 98 °C for 10 s, 50 °C for 15 s, and 68 °C for 15 s. PCR products were tagged using a Nextera XT Index Kit (Illumina, San Diego, CA, USA) to distinguish sample IDs using 10 ng DNA, Nextera XT Index Primer1, Nextera XT Index Primer2, 2× Gflex PCR Buffer (Mg^2+^, dNTP plus), and Tks Gflex DNA Polymerase (Takara Bio) in a volume of 25 µL at 94 °C for 1 min followed by eight cycles of 98 °C for 10 s, 60 °C for 15 s, and 68 °C for 15 s. Final PCR products were mixed to provide 2 ng of DNA for each sample. This was sent to Takara Bio for metagenomic analysis.

### 2.7. Short-Chain Fatty Acid (SCFA) and Gamma-Aminobutyric Acid (GABA) Analyses

SCFAs were derivatized and analyzed by liquid chromatography-tandem mass spectrometry (LC-MS/MS). Acetic, propionic, and butyric acid were quantified, and the results were expressed in μmol/g of wet weight cecum contents or μmol/L of serum. Details of the derivatization procedure and LC-MS/MS analysis of SCFAs were described by Zeng et al [12,26]. Serum GABA was determined using an ELISA kit (ImmuSmol SAS, Pessac, France) as described [12,27,28].

### 2.8. Statistical Analysis

Results are expressed as the mean ± standard error of the mean (SE). Data were analyzed by the one-way analysis of variance followed by the Tukey–Kramer post hoc test, if appropriate. All analyses were performed with a significance level of α = 0.05 (*p* < 0.05) using BellCurve for Excel (Social Survey Research Information Co., Ltd., Tokyo, Japan).

## 3. Results

### 3.1. Growth Parameters

Body weights, food intake and tissue weights of experimental animals are shown in Table 3. There was no significant difference in initial body weights among these groups. Final body weights increased significantly in the groups that were fed a high-fat diet (HD) compared to those in the normal diet (ND) group (HD vs. ND: *p* < 0.001). Food intake was significantly decreased in groups fed the high-fat diet compared to that in the ND group (HD vs. ND: *p* < 0.001). However, there was no significant difference in energy consumption among groups. Brain and heart weights were significantly decreased in the HD group compared to that in the ND group (*p* = 0.011 and 0.045, respectively). Moreover, there were no significant differences in brain and heart weights among the groups fed a high-fat diet. In addition, there were no significant differences in lung, liver, pancreas, spleen, or kidney weights among groups. Mesenteric adipose tissue weights were significantly decreased in the high-fat diet containing 1.0% Euglena and 0.3% vegetables (HDE + V) group compared to those in the HD group (*p* = 0.024). Perinephric and total white adipose tissue weights were significantly increased in the HD group compared to those in the ND group (*p* < 0.001 for both), and were significantly decreased in the high-fat diet containing 0.3% paramylon (HDP) group, the the high-fat diet containing 1.0% Euglena (HDE) group, and the HDE + V groups compared to that in the HD group (HDP vs. HD: *p* = 0.005 and 0.006, respectively; HDE vs. HD: *p* < 0.001 for both; HDE + V vs. HD: *p* < 0.001 for both). In addition, these values were significantly decreased in the HDE + V group compared to those in the HDP group (*p* < 0.001 and *p* = 0.028, respectively). Epididymal adipose tissue weight was significantly increased in the HD group compared to that in the ND group (*p* < 0.001), and was significantly decreased in the HDP, HDE, and HDE + V groups compared to that in the HD group, with the HDE + V group having the lowest white adipose tissue weight (HDP vs. HD: *P* = 0.031; HDE vs. HD: *p* < 0.001; HDE + V vs. HD: *p* < 0.001). These results show that visceral fat accumulation was suppressed through the ingestion of paramylon and Euglena, and that the simultaneous intake of Euglena and vegetables exerted a particularly strong effect.

### 3.2. Histological Analysis of Epididymal Adipose Tissue

**Figure 1:**
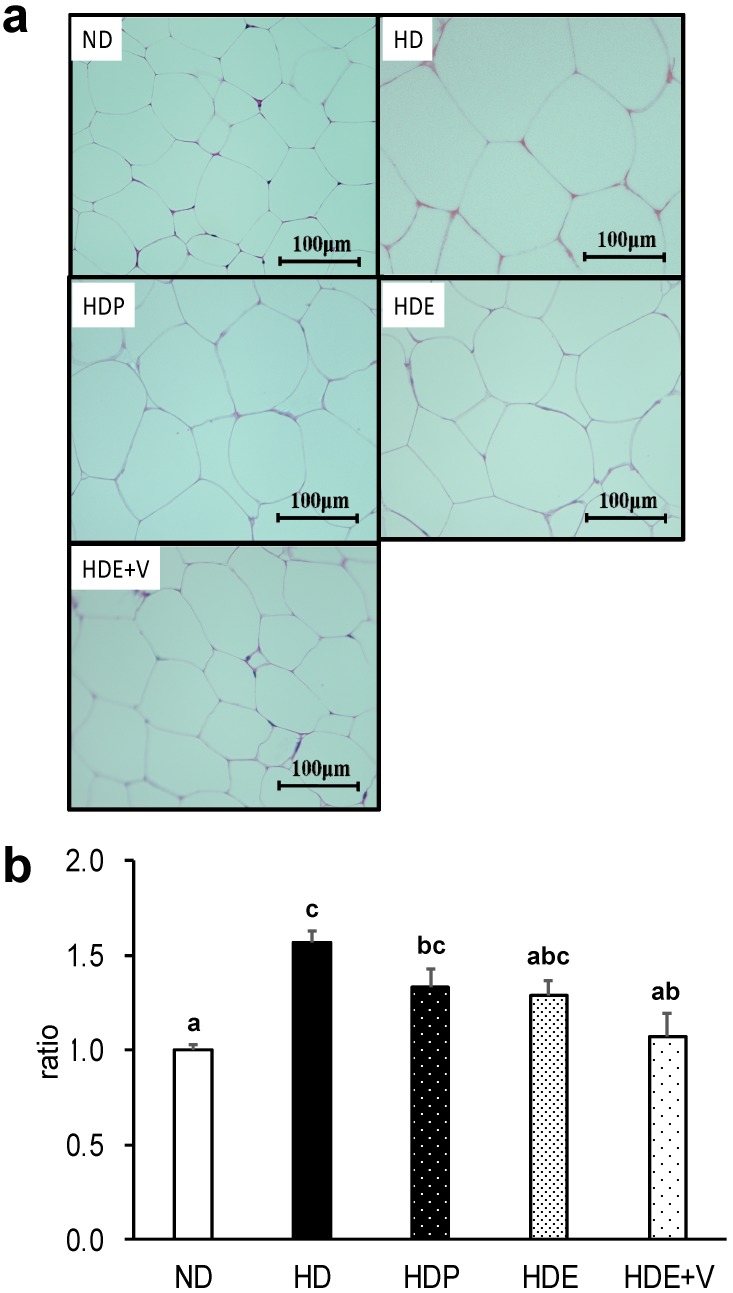
Effects of the intake of Euglena and vegetables on white adipose tissue in diet-induced obese mice. (**a**) Epididymal adipose tissue sections from representative mice in each group (hematoxylin & eosin, scale bar = 100 µm). (**b**) Adipocyte size ratio values are presented as the mean ± standard error of the mean, *n* = 10. ^a,b,c^ Different superscript letters indicate significantly different means at *p* < 0.05 (refer to “Result” in detail). ND, a group fed the normal diet; HD, a group fed the high-fat diet; HDP, a group fed the high-fat diet containing 0.3% paramylon; HDE, a group fed the high-fat diet containing 1.0% Euglena; HDE + V, a group fed the high-fat diet containing 1.0% Euglena and 0.3% vegetables.

The large changes in adipose tissue weight suggested that adipocyte sizes might differ among the groups. Histological observations of epididymal adipose tissue based on HE staining (Figure 1A) showed that adipocytes in the HD group were bigger than those in the ND group, but were smaller in the HDE + V group compared to the HD group. The adipocyte area was significantly increased in the HD group compared to the ND group (*p* < 0.001), but was significantly decreased in the HDE + V group compared to the HD group (*p* = 0.001; Figure 1B). Although the adipocyte area was marginally decreased following the intake of Euglena or paramylon, this effect did not reach statistical significance. These results show that the expansion of adipocytes was suppressed by Euglena consumption, with the simultaneous intake of Euglena and vegetables exerting a particularly strong effect.

### 3.3. mRNA Levels of Lipid Metabolism-Related Genes in Epididymal Adipose Tissue

The apparent suppression of visceral fat accumulation in the HDE + V group prompted us to examine the mRNA levels of lipid metabolism-related genes in epididymal adipose tissue, all of which play a central role in visceral fat accumulation (Table 4). mRNA levels of *Srebf1* (encoding a transcription factor for fatty acid synthesis pathway enzyme genes) was higher in the HD group than in the ND group, and was lower in the HDE and HDE + V groups than in the HD group (HD vs. ND: *p* = 0.034; HDE vs. HD: *p* = 0.009; HDE + V vs. HD: *p* = 0.003), which suggests that the intake of Euglena suppressed fatty acid synthesis in adipose tissues. mRNA levels of *Fasn* and *Me* (encoding fatty acid synthesis pathway enzymes) were higher in the HD group than in the ND group (*p* = 0.007 and 0.004, respectively). The HDE + V group expressed the lowest levels of these genes among the groups fed a high-fat diet, showing that the simultaneous intake of Euglena and vegetables suppressed the fatty acid synthesis system in adipose tissues. mRNA levels for *Pparg*, which encodes a protein that promotes cell division, were decreased in the HD group compared to those in the ND group (*p* = 0.037), with the HDE + V group exhibiting the highest levels among groups fed a high-fat diet. mRNA levels of *Hsl*, which encodes a protein that promotes lipolysis, were decreased in the HD group compared to those in the ND group (*p* = 0.009) and increased in the HDE + V group compared to those in the HD, HDP, and HDE groups (HDE + V vs. HD: *p* = 0.003; HDE + V vs. HDP: *p* = 0.004; HDE + V vs. HDE: *p* = 0.011, respectively), suggesting that the simultaneous intake of Euglena and vegetables promoted lipolysis in adipose tissues. There were no significant differences in *G6pdx* mRNA levels among the groups.

### 3.4. Biochemical Parameters in the Serum and Liver

**Figure 2:**
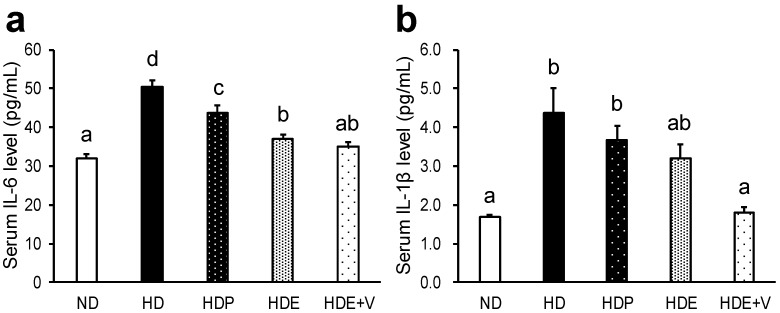
Effects of the intake of Euglena and vegetables on inflammation parameters in diet-induced obese mice. (**a**) Serum IL-6 level and (**b**) Serum IL-1β level are presented as the mean ± standard error of the mean, *n* = 10. ^a,b,c^ Different superscript letters indicate significantly different means at *p* < 0.05 (refer to “Result” in detail). ND, a group fed the normal diet; HD, a group fed the high-fat diet; HDP, a group fed the high-fat diet containing 0.3% paramylon; HDE, a group fed the high-fat diet containing 1.0% Euglena; HDE + V, a group fed the high-fat diet containing 1.0% Euglena and 0.3% vegetables.

Biochemical parameters in the serum and liver are shown in Figure 2 and Table 5. Serum IL-6, an index of inflammation, was significantly increased in the HD group compared to the ND group (*p* < 0.001) and was decreased in the HDP, HDE, and HDE + V groups compared to the HD group, with the HDE + V group having the lowest levels among groups fed a high-fat diet (HDP vs. HD: *p* = 0.002; HDE vs. HD: *p* < 0.001; HDE + V vs. HD: *p* < 0.001). In addition, these levels were decreased in the HDE and HDE + V groups compared to the HDP group (HDE vs. HDP: *p* = 0.002; HDE + V vs. HDP: *p* < 0.001). Serum IL-1β, another index of inflammation, was also significantly increased in the HD group compared to the ND group (*p* < 0.001) and this was decreased in the HDE + V group compared to the HD and HDP groups (HDE + V vs. HD: *p* < 0.001; HDE + V vs. HDP: *p* = 0.006, respectively). Serum TC and leptin were significantly increased in the HD group compared to the ND group (*p* = 0.011 and 0.046, respectively), with the HDE + V group having the lowest levels among groups fed a high-fat diet. Serum insulin was significantly increased in the HD group compared to the ND group (*p* = 0.008) and was decreased in the HDE + V group compared to the HD group (*p* = 0.049). Serum TBARS, an index of lipid peroxidation, was significantly increased in the HD group compared to the ND group (*p* = 0.041) and was decreased in the HDE + V group compared to the HD and HDE groups (HDE + V vs. HD: *p* = 0.022; HDE + V vs. HDE: *p* = 0.017). Liver TBARS was significantly lower in the HDE and HDE + V groups compared to the HD group (HDE vs. HD: *p* = 0.026; HDE + V vs. HD: *p* = 0.016). Although these parameters were marginally lower following the intake of paramylon, the effect of this diet did not reach statistical significance. Therefore, we concluded that inflammation and lipid peroxidation were suppressed by the simultaneous intake of Euglena and vegetables.

### 3.5. Gut Microbiota

The aforementioned results show that the simultaneous intake of Euglena and vegetables had a greater effect than consumption of Euglena only. To clarify the factors involved in these effects, changes in the gut microbiota of the HDE + V group were examined. Homology searches and lineage classification analyses were performed using the 16S rRNA database with obtained sequences, and the genera of microorganisms in the samples were determined. Detailed analysis identified 13 taxonomic units for which the proportion was at least two-fold higher or lower in the HD group compared to that in the ND group (Table 6). *Sporosarcina*, *Bacteria;Other;Other;Other;Other;Other*, *Lactococcus*, *Anaerotruncus*, *Clostridiaceae;Other*, and *Coprobacillus* were two-fold more abundant in the HD group. Furthermore, *Erysipelotrichaceae;Other*, *Bifidobacterium*, *Lactobacillus*, *F16;g__*, *Clostridia;Other;Other;Other*, *Enterococcaceae;Other*, and *Escherichia* were ≥ 2-fold less frequent in the HD group. It was not possible to determine the genus of *F16;g__*. *Sporosarcina*, *Bacteria;Other;Other;Other;Other;Other*, *Lactococcus*, and *Clostridiaceae;Other* were increased in the HD group compared to the ND group, but were decreased in the HDP group compared to the HD group, decreased in the HDE group compared to the HDP group, and decreased in the HDE + V group compared to the HDE group. Furthermore, *Erysipelotrichaceae;Other*, *Bifidobacterium*, *Lactobacillus*, *F16;g__*, and *Clostridia;Other;Other;Other* were decreased in the HD group compared to the ND group, increased in the HDP group compared to the HD group, increased in the HDE group compared to the HDP group, and increased in the HDE + V group compared to the HDE group. These differences in gut microbiota composition caused by the simultaneous intake of Euglena and vegetables were likely underlying factors of the different effects of diets on physiological functions. 

### 3.6. SCFAs and GABA Levels in Cecum Content and Serum

Given that the gut microbiota was substantially changed due to the simultaneous intake of Euglena and vegetables, levels of SCFAs and GABA, for which the production is promoted by intestinal bacteria, were measured (Table 7). Acetic, propionic, and butyric acids levels in the cecum content were significantly decreased in the HD group compared to those in the ND group, with the HDE + V group having the highest levels among groups fed a high-fat diet (HD vs. ND, *p* = 0.031, *p* < 0.001 and *p* < 0.001, respectively). In addition, propionic acid levels were increased in the HDE + V group compared to the HD and HDP groups (HDE + V vs. HD: *p* < 0.001; HDE + V vs. HDP: *p* = 0.041). Although the SCFA levels showed were marginally higher following the intake of paramylon, this effect was not statistically significant. Serum acetic, propionic, and butyric acid levels were significantly decreased in the HD group compared to the ND group, with the HDE + V group having the highest levels among groups fed a high-fat diet (HD vs. ND, *p* < 0.001, 0.001, and 0.001, respectively). In addition, acetic acid levels were increased in the HDP, HDE, and HDE + V groups compared to the HD group (HDP vs. HD: *p* = 0.021; HDE vs. HD: *p* = 0.018; HDE + V vs. HD: *p* = 0.001). Propionic acid levels were increased in the HDE + V group compared to the HD, HDP, and HDE groups (HDE + V vs. HD: *p* < 0.001; HDE + V vs. HDP: *p* = 0.002, HDE + V vs. HD: *p* = 0.012). Butyric acid levels were higher in the HDE and HDE + V groups than in the HD and HDP groups (HDE vs. HD: *p* < 0.001; HDE vs. HDP: *p* < 0.001; HDE + V vs. HD: *p* < 0.001; HDE + V vs. HDP: *p* < 0.001). Serum GABA levels were significantly higher in the HDE + V group than in the HD group (*p* < 0.015). These results show that the production of SCFAs and GABA was promoted by the simultaneous intake of Euglena and vegetables.

## 4. Discussion

In this study, for which the objective was to increase the nutritional value of insoluble fiber-rich Euglena, we determined whether the consumption of soluble fiber-rich vegetables could augment its anti-obesity effect. Results showed that this was the case, with the consumption of paramylon and Euglena resulting in a significant decrease of visceral fat in obese mice. Histological observation of white adipose tissue and the analysis of gene expression related to lipid metabolism showed that the simultaneous consumption of Euglena and vegetables decreased adipocyte area, suppressed gene expression related to fatty acid synthesis, and increased gene expression related to adipocyte lipolysis. We also observed a decrease in serum inflammation parameters. To explore the associated mechanisms, we examined the effect of Euglena on the intestinal microbiota, and observed the following: (1) Increases in beneficial bacteria (which were decreased due to obesity) that produce short-chain fatty acids and in beneficial bacteria that promote vitamin synthesis; (2) a decrease in harmful bacteria that cause inflammation, which had increased due to obesity; (3) an increase in short-chain fatty acids levels. Therefore, we confirmed that the simultaneous consumption of vegetables enhanced the anti-inflammatory effect of Euglena and its ability to suppress visceral fat accumulation; results suggested that changes in intestinal microbiota were involved in this effect. 

The final body weight of obese mice increased significantly due to high fat intake, but paramylon or Euglena intake did not affect this. Moreover, due to the high-fat diet intake, the amount of feed administered was significantly decreased, but there was no difference in caloric intake between the groups. Thus, these changes likely originated from the high-fat diet. However, there was a decrease in the weight of white adipose tissue due to paramylon and Euglena intake, and in the case of perinephric and epididymal adipose tissue, this change was particularly pronounced. In studies using rat models of type 2 diabetes, visceral fat accumulation was inhibited by a diet including paramylon and Euglena, with the latter being more effective [3,5]. This difference was believed to be due to various beneficial components in Euglena in addition to paramylon. The same effect was observed in this study, and the effect of Euglena was further enhanced by vegetables. We previously demonstrated that one of the reasons why the traditional Japanese diet is more beneficial for preserving health than the contemporary diet is that it is important to consume a variety of food components [14,29,30,31]. Therefore, consuming small amounts of a variety of food components is extremely effective for preserving health, which suggests that the joint effect of Euglena and vegetables increased due to the diversity of food components.

Histological observation of epididymal adipose tissue showed that the adipocyte area exhibited a similar trend to visceral fat amounts. In addition, the expression of *Fasn*, *Me*, and *Srebp-1c* [32], which encode molecules that promote fat accumulation, exhibited the same trend as visceral fat levels. Meanwhile, the expression of *Ppar γ* [33], which promotes adipocyte proliferation and induces adipocyte miniaturization, and *Hsl* [34], which induces lipolysis and adipocyte miniaturization, exhibited the opposite trend. These findings demonstrated that the combined use of vegetables enhanced the effects of Euglena.

In this study, serum levels of IL-1β and IL-6 [35], which are parameters of inflammation, decreased with Euglena consumption and decreased further when vegetables were added. This demonstrated that vegetables enhanced the anti-inflammatory effects of Euglena [3]. Since maintaining low levels of inflammatory markers prevents the onset of aging-associated diseases and is thus useful for preserving health [35], this suggests that the joint consumption of Euglena and vegetables could be beneficial for health and longevity. This potential was further suggested by the fact that the consumption of both Euglena and vegetables led to a significant decrease in the amount of over-oxidized fat and a reduction in oxidative stress.

To examine the mechanism associated with the anti-obesity and anti-inflammatory effects of Euglena, we investigated its effect on intestinal microbiota. Genera that increased with the consumption of a high-fat diet included *Sporosarcina*, *Bacteria;Other;Other;Other;Other;Other*, *Lactococcus*, *Anaerotruncus*, *Clostridiaceae;Other*, and *Coprobacillus*. In this study, *Sporosarcina*, *Bacteria;Other;Other;Other;Other;Other*, *Lactococcus*, and *Clostridiaceae;Other* increased with a high-fat diet, but decreased with paramylon consumption, decreased further with Euglena consumption, and decreased even further with the consumption of both Euglena and vegetables. This is a similar tendency to that observed for visceral fat. Levels of *Sporosarcina* were previously found to increase when hepatitis was induced by artificial sweeteners [36]. Therefore, increases in these genera might promote inflammation. Lactococcus includes useful bacteria that produce lactic acid but also putrefying bacteria [37]. The function of *Bacteria;Other;Other;Other;Other;Other* has not yet been determined, whereas *Clostridiaceae;Other* includes harmful bacteria that cause food poisoning [38]. Here, harmful bacteria such as those increased with obesity were inhibited with the consumption of paramylon, Euglena, and vegetables. In addition, *Coprobacillus* functions to inhibit the proliferation of the toxin-producing bacterium *Clostridium difficile* [39], although we did not observe any major changes in this species due to paramylon or Euglena consumption. *Anaerotruncus* produces short-chain fatty acids such as butyric acid and functions to inhibit inflammation [40], and this experiment confirmed that paramylon and Euglena consumption could increase this genus. 

Genera that decreased greatly with the consumption of a high-fat diet included *Erysipelotrichaceae;Other*, *Bifidobacterium*, *Lactobacillus*, *F16;g__*, *Clostridia;Other;Other;Other*, *Enterococcaceae;Other*, and *Escherichia*. In this study, *Erysipelotrichaceae;Other*, *Bifidobacterium*, *Lactobacillus*, *F16;g__*, and *Clostridia;Other;Other;Other* decreased with the high-fat diet, increased with paramylon consumption, increased further with Euglena consumption, and increased even further with the consumption of both Euglena and vegetables. This represented a trend to that observed for visceral fat amounts. *Erysipelotrichaceae;Other* has been reported to increase with β-glucan and inulin consumption and is involved in the production of short-chain fatty acids [41]. In this study, this group increased with the consumption of soluble fiber-rich vegetables, as did the short-chain fatty acid levels. Short-chain fatty acids exert an anti-inflammatory effect by strengthening the intestinal barrier function and an anti-obesity effect by increasing energy expenditure in the liver [41,42]. Consequently, this suggests that the effect of vegetables was due to increasing levels of short-chain fatty acids. *Bifidobacterium* metabolizes sugars in the intestine to produce acetic acid and lactic acid [43]. Acetic acid, which is a short-chain fatty acid, was also found to increase with the consumption of both Euglena and vegetables. *Lactobacillus* metabolizes sugars in the intestine to produce lactic acid and encourages the production of GABA and other useful substances [44,45]. Lactic acid inhibits the growth of harmful bacteria that cannot survive in acidic conditions and exerts an anti-inflammatory effect. In this study, we observed that the consumption of Euglena and vegetables resulted in an increase in GABA, which possesses stress-reducing properties [27,45]. The function of *F16;g__* has not yet been determined. *Clostridia;Other;Other;Other* includes harmful bacteria such as *Clostridrium perfringes* [46] and *Clostridrium difficile* [47], which cause contagious diseases. We did not find any remarkable changes in these species based on the consumption of paramylon or Euglena. In our experiments, obesity suppressed levels of beneficial bacteria, whereas the consumption of paramylon, Euglena, and vegetables promoted their abundance. In addition, *Enterococcaceae;Other*, which produces lactic acid [48], was scarcely detected in the high-fat diet groups of this study. *Escherichia* encompasses many bacteria including *Escherichia coli*. *E. coli* is decreased in individuals who consume plenty of fiber [49]. In this study, this species decreased with the consumption of paramylon and Euglena. Based on these data, we showed that the joint consumption of Euglena and vegetables resulted in an increase in beneficial bacteria and a decrease in harmful bacteria, which we believe was one of the mechanisms associated with the beneficial effects of vegetables.

## 5. Conclusions

Our findings confirmed that the simultaneous consumption of vegetables enhanced the anti-obesity and anti-inflammatory effects of Euglena and suggested that intestinal microbiota are involved in this effect. Due to its various nutritional and beneficial effects, Euglena has the potential to be an important food source for society in the future. It is thus crucial to establish more efficient ways to consume Euglena. We will perform further research to confirm its effects in humans and discover more efficient ways to consume this product.

## Figures and Tables

**Table 1:** Composition of test diets.

	Normal	High-Fat	High-Fat + Paramylon	High-Fat + Euglena	High-Fat + Euglena + Vegetables
	diet	diet	diet	diet	diet
Ingredient	(g/100g diet)				
Casein	16.3	19.5	19.5	19.3	19.3
DL-Methionine	0.3	0.3	0.3	0.3	0.3
Corn Starch	33.8	5.0	4.9	4.7	4.5
Maltodextrin 10	8.4	10.0	10.0	9.9	9.9
Sucrose	28.5	34.1	34.0	33.8	33.7
Cellulose	4.2	5.0	5.0	5.0	4.9
Corn Oil	4.4	1.0	1.0	1.0	0.9
Milk Fat, Anhydrous	-	20.0	20.0	20.0	20.0
Mineral Mix (S10001)	2.9	3.5	3.5	3.5	3.5
Calcium Carbonate	0.3	0.4	0.4	0.4	0.4
Vitamin Mix (V10001)	0.8	1.0	1.0	1.0	1.0
Choline Bitartrate	0.2	0.2	0.2	0.2	0.2
Cholesterol	-	0.2	0.2	0.2	0.2
Paramylon	-		0.3	-	-
Euglena	-		-	1.0	1.0
Barley leaf	-		-	-	0.1
Kale	-		-	-	0.1
Ashitaba	-		-	-	0.1
Others	-		-	-	0.1
	(energy %)				
Protein	17.1	17.0	17.0	17.0	17.1
Carbohydrate	72.7	42.2	42.2	42.0	41.9
Fat	10.2	40.7	40.8	41.0	41.0
Energy (kcal/100g)	389	465	464	461	459

High-fat + paramylon diet, the high-fat diet containing 0.3% paramylon; high-fat + *Euglena gracilis* (Euglena) diet, the high-fat diet containing 1.0% Euglena; high-fat + Euglena + vegetables diet, the high-fat diet containing 1.0% Euglena and 0.3% vegetables.

**Table 2:** Primer pairs used for the real time qRT-PCR analysis.

Genbank ID	Gene Name		Primer Sequence (5′ to 3′)
NM_007988	*Fasn*	Forward	CCTGGATAGCATTCCGAACCTG
		Reverse	TTCACAGCCTGGGGTCATCTTTGC
NM_008062	*G6pdx*	Forward	TGGGTCCACCACTGCCACTTTTG
		Reverse	ATTGGGCTGCACACGGATGACCA
NM_001039507	*Hsl*	Forward	TTCTCCAAAGCACCTAGCCAA
		Reverse	TGTGGAAAACTAAGGGCTTGTTG
M29546	*Me*	Forward	GAAAGAGGTGTTTGCCCATGA
		Reverse	AATTGCAGCAACTCCTATGAGG
NM_011146	*Ppar γ*	Forward	GGAAGACCACTCGCATTCCTT
		Reverse	TCGCACTTTGGTATTCTTGGAG
NM_011480	*Srebf1*	Forward	GGAGACATCGCAAACAAGC
		Reverse	TGAGGTTCCAAAGCAGACTG
NM_007393	*Actb*	Forward	GGCTGTATTCCCCTCCATCG
		Reverse	CCAGTTGGTAACAATGCCATGT

*Fasn*, fatty acid synthase; *G6pdx*, glucose-6-phosphate 1-dehydrogenase X; *Hsl*, hormone sensitive lipase; *Me*, malic enzyme; *Ppar γ*, peroxisome proliferator-activated receptor gamma; *Srebf1*, sterol regulatory element-binding protein 1c; *Actb*, actin beta.

**Table 3:** Body weights, food intake and tissue weights.

	ND	HD	HDP	HDE	HDE + V
Initial body weight (g)	22.9 ± 0.3	22.9 ± 0.3	22.9 ± 0.3	22.9 ± 0.2	22.9 ± 0.2
Final body weight (g)	29.4 ± 0.6 ^a^	33.8 ± 0.8 ^b^	33.4 ± 0.6 ^b^	32.8 ± 0.7 ^b^	32.9 ± 0.5 ^b^
Food intake (g/day)	3.01 ± 0.04 ^a^	2.62 ± 0.03 ^b^	2.63 ± 0.05 ^b^	2.68 ± 0.04 ^b^	2.59 ± 0.04 ^b^
Energy intake (kcal/day)	11.7 ± 0.2	12.2 ± 0.1	12.2± 0.2	12.4 ± 0.2	11.9 ± 0.2
Tissue weight (g/100 g body weight)					
Brain	1.57 ± 0.04 ^b^	1.41 ± 0.04 ^a^	1.42 ± 0.02 ^a^	1.44 ± 0.03 ^a,b^	1.45 ± 0.03 ^a,b^
Heart	0.45 ± 0.01 ^b^	0.40 ± 0.01 ^a^	0.42 ± 0.01 ^a,b^	0.43 ± 0.01 ^a,b^	0.44 ± 0.01 ^a,b^
Lung	0.76 ± 0.05	0.78 ± 0.08	0.86 ± 0.06	0.87 ± 0.11	0.76 ± 0.08
Liver	3.31 ± 0.03	3.42 ± 0.06	3.36 ± 0.05	3.33 ± 0.06	3.39 ± 0.04
Pancreas	0.68 ± 0.03	0.64 ± 0.03	0.65 ± 0.03	0.68 ± 0.02	0.69 ± 0.04
Spleen	0.29 ± 0.02	0.27 ± 0.01	0.31 ± 0.02	0.27 ± 0.01	0.29 ± 0.01
Kidney	1.10 ± 0.03	1.04 ± 0.02	1.11 ± 0.03	1.08 ± 0.03	1.01 ± 0.02
White adipose tissue					
Mesenteric	1.29 ± 0.05 ^a,b^	1.38 ± 0.09 ^b^	1.21 ± 0.12 ^a,b^	1.17 ± 0.06 ^a,b^	1.02 ± 0.08 ^a^
Perinephric	1.38 ± 0.10 ^a^	1.98 ± 0.10 ^c^	1.55 ± 0.06 ^b^	1.34 ± 0.07 ^a,b^	1.05 ± 0.08 ^a^
Epididymal	2.61 ± 0.15 ^a^	3.92 ± 0.18 ^b^	3.05 ± 0.23 ^a^	2.64 ± 0.27 ^a^	2.48 ± 0.15 ^a^
Total	5.28 ± 0.28 ^a^	7.28 ± 0.34 ^c^	5.81 ± 0.35 ^b^	5.15 ± 0.22 ^a,b^	4.56 ± 0.23 ^a^

Values are mean ± SE, *n* = 10. ^a,b,c^ Different superscript letters indicate significantly different means at *p* < 0.05. ND, a group fed the normal diet; HD, a group fed the high-fat diet; HDP, a group fed the high-fat diet containing 0.3% paramylon; HDE, a group fed the high-fat diet containing 1.0% Euglena; HDE + V, a group fed the high-fat diet containing 1.0% Euglena and 0.3% vegetables.

**Table 4:** mRNA expression level in white adipose tissue (ratio).

	ND	HD	HDP	HDE	HDE + V	Gene Function
*Fasn*	1.00 ± 0.10 ^a^	1.77 ± 0.17 ^b^	1.81 ± 0.24 ^b^	1.72 ± 0.05 ^b^	1.29 ± 0.13 ^a,b^	Fatty acid synthesis
*G6pdx*	1.00 ± 0.14	1.42 ± 0.10	1.34 ± 0.11	1.27 ± 0.08	1.10 ± 0.14	
*Me*	1.00 ± 0.06 ^a^	1.65 ± 0.15 ^b,c^	1.70 ± 0.07 ^c^	1.58 ± 0.15 ^b,c^	1.20 ± 0.12 ^a,b^	
*Srebp-1c*	1.00 ± 0.11 ^a^	1.49 ± 0.13 ^b^	1.28 ± 0.10 ^a,b^	0.90 ± 0.14 ^a^	0.86 ± 0.10 ^a^	
*Ppar γ*	1.00 ± 0.06 ^a^	0.79 ± 0.06 ^b^	0.69 ± 0.03 ^b^	0.75 ± 0.04 ^b^	0.89 ± 0.07 ^a,b^	Cell division
*Hsl*	1.00 ± 0.06 ^a^	0.54 ± 0.06 ^b^	0.56 ± 0.07 ^b^	0.60 ± 0.09 ^b^	1.05 ± 0.15 ^a^	Lipolysis

Values are mean ± SE, *n* = 10. ^a,b,c^ Different superscript letters indicate significantly different means at *p* < 0.05. ND, a group fed the normal diet; HD, a group fed the high-fat diet; HDP, a group fed the high-fat diet containing 0.3% paramylon; HDE, a group fed the high-fat diet containing 1.0% Euglena; HDE + V, a group fed the high-fat diet containing 1.0% Euglena and 0.3% vegetables.

**Table 5:** Biochemical parameter in serum and liver.

	ND	HD	HDP	HDE	HDE + V
**Serum**					
TG (mmol/L)	1.38 ± 0.08	1.07 ± 0.10	1.14 ± 0.12	1.25 ± 0.07	1.18 ± 0.06
TC (mmol/L)	3.29 ± 0.15 ^a^	4.31 ± 0.12 ^b^	4.07 ± 0.22 ^a,b^	4.01 ± 0.15 ^a,b^	3.50 ± 0.35 ^a,b^
PL (mmol/L)	73.9 ± 2.7	78.5 ± 2.7	72.5 ± 6.7	84.8 ± 2.3	83.1 ± 2.3
Glucose (mmol/L)	5.55 ± 0.27	7.03 ± 0.50	5.87 ± 0.47	6.44 ± 0.29	6.95 ± 0.33
Insulin (μg/L)	0.19 ± 0.01 ^a^	0.27 ± 0.01 ^c^	0.26 ± 0.02 ^b,c^	0.23 ± 0.02 ^a,b,c^	0.21 ± 0.01 ^a,b^
TBARS (µmol/L)	7.16 ± 0.33 ^a^	6.12 ± 0.24 ^b^	5.27 ± 0.26 ^b,c^	6.12 ± 0.22 ^b^	4.98 ± 0.15 ^c^
ALT (IU/L)	5.03 ± 0.41	6.17 ± 0.70	4.77 ± 0.43	4.87 ± 0.46	6.20 ± 0.64
AST (IU/L)	54.6 ± 3.3	67.5 ± 10.6	61.6 ± 8.7	58.4 ± 6.3	63.0 ± 7.8
Leptin (ng/mL)	4.27 ± 0.69 ^a^	8.99 ± 1.43 ^b^	7.61 ± 1.19 ^a,b^	7.62 ± 1.20 ^a,b^	6.60 ± 0.79 ^a,b^
**Liver**					
TG (μmol/g)	43.2 ± 7.1	53.6 ± 4.2	48.6 ± 6.3	41.0 ± 7.1	38.3 ± 5.5
TC (μmol/g)	8.59 ± 1.17	10.3 ± 1.2	10.1 ± 1.8	9.14 ± 1.30	8.56 ± 0.99
PL (μmol/g)	35.2 ± 0.6	35.9 ± 1.0	36.1 ± 0.4	36.2 ± 0.9	35.0 ± 0.7
TBARS (nmol/g)	53.1 ± 3.2 ^a,b^	54.8 ± 2.4 ^b^	49.6 ± 1.1 ^a,b^	45.4 ± 1.6 ^a^	45.1 ± 1.4 ^a^

Values are mean ± SE, *n* = 10. ^a,b,c^ Different superscript letters indicate significantly different means at *p* < 0.05. ND, a group fed the normal diet; HD, a group fed the high-fat diet; HDP, a group fed the high-fat diet containing 0.3% paramylon; HDE, a group fed the high-fat diet containing 1.0% Euglena; HDE + V, a group fed the high-fat diet containing 1.0% Euglena and 0.3% vegetables.

**Table 6:** Gut microbiota (genus level) that increased by more than two times or decreased to 1/2 or less in the HD group compared to the ND group.

	ND	HD	HDP	HDE	HDE + V	HD/ND
*Sporosarcina (%)*	0.00	1.47	0.64	0.21	0.01	368
*Bacteria;Other;Other;Other;Other;Other (%)*	0.07	0.84	0.14	0.28	0.58	12.0
*Lactococcus (%)*	0.08	0.51	0.45	0.21	0.17	6.38
*Anaerotruncus (%)*	0.06	0.35	0.43	0.56	0.8	5.83
*Clostridiaceae;Other (%)*	1.01	2.49	1.52	1.02	0.38	2.47
*Coprobacillus (%)*	0.01	0.02	0.02	0.02	0.03	2.00
*Erysipelotrichaceae;Other (%)*	0.03	0.01	0.02	0.03	0.05	0.33
*Bifidobacterium (%)*	11.3	1.46	2.46	5.79	10.0	0.13
*Lactobacillus (%)*	2.51	0.23	1.31	2.27	4.22	0.09
*F16;g__ (%)*	0.51	0.04	0.04	0.08	0.15	0.08
*Clostridia;Other;Other;Other (%)*	0.54	0.04	0.11	0.57	0.28	0.07
*Enterococcaceae;Other (%)*	0.25	0.01	0.00	0.00	0.00	0.04
*Escherichia (%)*	14.2	0.06	0.03	0.03	0.02	0.00

**Table 7:** SCFAs and GABA levels in cecum content and serum.

	ND	HD	HDP	HDE	HDE + V
**Cecum content**					
Acetic acid (μmol/g)	37.5 ± 4.2 ^b^	25.8 ± 2.5 ^a^	28.4 ± 2.1 ^a,b^	29.8 ± 2.8 ^a,b^	35.1 ± 1.1 ^a,b^
Propionic acid (μmol/g)	16.9 ± 0.5 ^b,c^	12.6 ± 0.6 ^a^	14.6 ± 0.7 ^a,b^	15.5 ± 0.7 ^b,c^	17.4 ± 0.9 ^c^
Butyric acid (μmol/g)	9.90± 0.81 ^b^	3.36 ± 0.72 ^a^	5.07 ± 1.26 ^a^	5.94 ± 1.05 ^a,b^	6.42 ± 1.20 ^a,b^
**Serum**					
Acetic acid (μmol/L)	400 ± 11 ^c^	314 ± 8 ^a^	358 ± 10 ^b^	359 ± 8 ^b^	371 ± 11 ^b,c^
Propionic acid (μmol/L)	5.08 ± 0.14 ^b^	3.78 ± 0.18 ^a^	3.91 ± 0.18 ^a^	4.06 ± 0.17 ^a^	4.86 ± 0.17 ^b^
Butyric acid (μmol/L)	3.31 ± 0.09 ^b^	2.38 ± 0.13 ^a^	2.42 ± 0.13 ^a^	3.23 ± 0.12 ^b^	3.43 ± 0.10 ^b^
GABA (μmol/L)	6.85 ± 0.28 ^a,b^	5.87 ± 0.15 ^a^	6.02 ± 0.18 ^a^	6.86 ± 0.32 ^ab^	7.07 ± 0.31 ^b^

Values are mean ± SE, *n* = 10. ^a,b,c^ Different superscript letters indicate significantly different means at *p* < 0.05. ND, a group fed the normal diet; HD, a group fed the high-fat diet; HDP, a group fed the high-fat diet containing 0.3% paramylon; HDE, a group fed the high-fat diet containing 1.0% Euglena; HDE + V, a group fed the high-fat diet containing 1.0% Euglena and 0.3% vegetables.
